# Association of Duration of Smoking Cessation or Cumulative Smoking Amount with Serum hs-CRP Level in Korean Adults: A Nationwide Population-Based Cross-Sectional Study

**DOI:** 10.3390/toxics10090533

**Published:** 2022-09-10

**Authors:** Ju-Hye Cho, Duk-Chul Lee, Hye-Jun Lee

**Affiliations:** 1 Department of Family Medicine, College of Medicine, Yonsei University, Seoul 03722, Korea; 2 Department of Family Medicine, Chung-ang University Hospital, College of Medicine, Chung-ang University, Seoul 06973, Korea

**Keywords:** duration of smoking cessation, cumulative smoking amount, serum hs-CRP, former smokers

## Abstract

This study investigates the association between the duration of smoking cessation or cumulative smoking amount with serum high-sensitivity C-reactive protein (hs-CRP) levels. We assessed the decreasing risk for cardiovascular disease (CVD) and cancer following smoking cessation in Korean adults who were former smokers compared with current smokers. This study used data from the 2016–2018 Korea National Health and Nutrition Examination Survey. A total of 5411 participants were included. The duration of smoking cessation and cumulative smoking amount were classified into cut-offs for 6 and 17 months, and 5 and 20 pack-years, respectively, using tertile values. Elevated serum hs-CRP level was defined as ≥1 mg/L. Multivariate logistic regression analysis was performed. The odds ratio (OR) for elevated serum hs-CRP level was 0.73 times lower in the group whose duration of smoking cessation was 17 months or more than that in the group who were current smokers after adjusting for confounding variables (95% confidence interval (CI): 0.57–0.92; *p* < 0.01). The OR for elevated serum hs-CRP level was 0.71 and 0.67 times lower in the groups whose cumulative smoking amounts were less than 5 and 5–20 pack-years than that in the group who were current smokers (95% CI: 0.50–0.99 and 0.50–0.92, respectively; both *p* < 0.05). This study reveals that a duration of smoking cessation of more than 17 months and a cumulative smoking amount of less than 20 pack-years were significantly associated with a decreased risk of elevated serum hs-CRP levels in Korean adults who were former smokers. Therefore, quitting smoking early and a low cumulative smoking amount are a potential preventive strategy for CVD and cancer that can be easily accessible using serum hs-CRP.

## 1. Introduction

Smoking is closely related to cardiovascular diseases (CVD) [1,2,3,4], and cancer [5,6]. In particular, the effect of smoking on CVD is significant. In the United States, one-third of CVD-related deaths are related to smoking [1]. Globally, 10% of all types of CVD are associated with smoking [2]. Smoking causes the chronic inflammation of blood vessels [7], leading to atherosclerosis [3]. As a result, it causes micro- and macrovascular complications, cancer, and sudden death [4]. As an indicator of chronic inflammation, high sensitivity-C reactive protein (hs-CRP) is now studied [8,9].

Serum hs-CRP is an acute reactive substance primarily synthesized by the liver and induced by inflammatory cytokines [10]. It increases acutely due to infectious diseases, noninfectious inflammatory responses, and other processes by a chain inflammatory signal; hence, it is clinically used to reflect the degree of infection and inflammation [11]. Several studies have shown that elevated hs-CRP, which is measurable at lower concentrations than CRP is in arteriosclerosis, implies the low-grade inflammation of the walls of blood vessels. This, in turn, indicates the likelihood of future morbidity to cardiovascular disease [8]. There are also several studies on the association of hs-CRP with CVD risk factors other than smoking (cholesterol, triglyceride, higher low-density lipoprotein cholesterol, lower high-density lipoprotein cholesterol [8,12], alcohol consumption [13], and obesity [8,14]). hs-CRP levels in the blood are also associated with the risk of developing cancers involving the breast, colon, and rectum [15,16,17,18,19].

Many studies were conducted on elevated serum hs-CRP concentrations in smokers [20,21,22]. Other studies attempted to predict the risk of CVD and explain the mechanisms of chronic vascular inflammation associated with the coagulation mechanism and lipid metabolism, which are promoted by smoking [20,23,24]. However, reductions in hs-CRP concentrations after smoking cessation remain controversial. While several studies showed that quitting smoking lowers the incidence of CVD and cancer compared to persistent smoking [25,26], data are conflicting regarding the hs-CRP of former smokers compared to that of current smokers. Relevant studies showed that quitting smoking reduces hs-CRP in those who quit smoking for longer than 5 to 20 years [27,28,29,30,31]; however, some studies showed no significant change in smoking cessation and hs-CRP reduction [32,33,34]. Additionally, studies on the reduction in hs-CRP levels after smoking cessation compared to current smokers are insufficient in the Korean population. There was a cross-sectional study of male Korean former smokers, but women were excluded, and only former smokers were compared in that study [35].

Since quitting smoking has a positive effect on reducing the incidence of CVD and cancer [36,37,38], the concentration of hs-CRP in the blood is also expected to decrease. If the baseline hs-CRP level in the blood, which is used as a predictor of CVD and cancer development, is reduced with smoking cessation, the direct benefits of disease prevention and the reduction in chronic intravascular inflammation can be visibly confirmed using serum hs-CRP, which can easily be obtained from a simple laboratory test.

Therefore, we hypothesized that, in former smokers compared to current smokers, there would be an association among a decrease in serum hs-CRP level, long periods of smoking cessation, and small cumulative amounts of smoking with serum hs-CRP level reduction. This study uses data from the Korean National Health and Nutrition Examination Survey (KNHANES) 2016–2018 to analyze the association between serum hs-CRP levels according to the duration of smoking cessation and cumulative smoking volume of former smokers compared with those of current smokers. 

## 2. Materials and Methods

### 2.1. Study Population

This cross-sectional study uses data from the 2016–2018 KNHANES. KNHANES is a nationwide population-based survey examining the general health and nutritional status of the civilian noninstitutionalized population in South Korea, conducted by the Korean Ministry of Health and Welfare, and the Division of Chronic Disease Surveillance of the Korean Centers for Disease Control and Prevention [39]. KNHANES uses a stratified, multistage, clustered probability sampling method for selecting a representative sample of the Korean population. Therefore, the statistical analyses in this study were conducted by adopting stratification, clustering, and weight variables [40,41]. Of the 24,269 people surveyed, 14,789 adults aged ≥ 19 years and <65 years were included. In Korea, minors (who are under 19 years old) tend to be extremely reluctant to disclose their smoking to their parents, and it is illegal for people who are underage to smoke. Therefore, in the household survey KNHANES, there is a high possibility that minors give false information about their smoking to interviewers. Therefore, we excluded minors because of the risk of bias. In the case of over-65-year-olds, old age is one of the factors of elevated CRP [42,43], so it is highly likely that their hs-CRP levels does not show a pattern on par with that of the younger population. So, we excluded old participants.

Participants with a white blood cell count ≥10,000/UI or hs-CRP > 3.0 mg/L, which may have been accompanied by acute infection, were excluded. Shine et al. reported that about 90% of normal healthy individuals showed a CRP level of less than 3 mg/L [44]. Mosby’s diagnostic and laboratory test reference distinguished between CRP and hs-CRP and mentions normal values were less than 10 mg/L for CRP and 3 mg/L for hs-CRP [45]. In participants with hs-CRP levels over 3 mg/L, inflammation and an early phase of acute infection cannot be ruled out. Therefore, we set less than 3 mg/L as the upper limit of normal.

**Figure 1:**
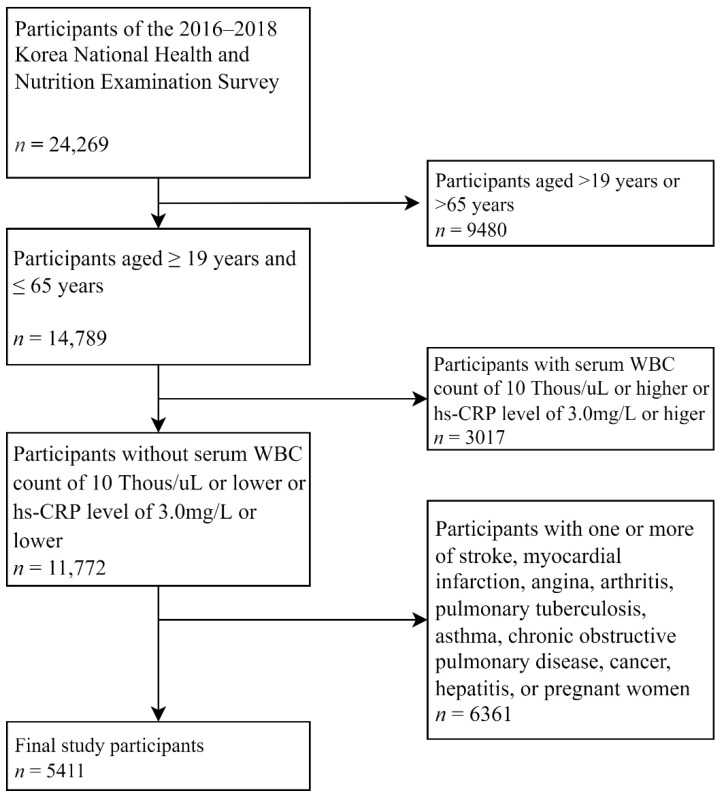
Flow diagram of the selection of study participants. WBC, white blood cell; hs-CRP, high sensitivity C-reactive protein.

Participants with a history of stroke, myocardial infarction, or angina pectoris; other diseases that may affect elevated hs-CRP levels, such as arthritis, pulmonary tuberculosis, asthma, chronic obstructive pulmonary disease, cancer, and hepatitis; and pregnant women were excluded from this study. We included 5411 participants (Figure 1).

All participants provided written informed consent before participating in the study [39]. KNHANES was conducted with ethical approval from the Institutional Review Board of the Korea Centers for Disease Control and Prevention (KNHANES was exempt from research ethics review on the basis of the Bioethics and Safety Act 2016–2017 [41], and no. 2018-01-03-P-A in 2018).

### 2.2. Data Collection

Trained staff interviewed the participants using standardized health questionnaires collecting data on demographic information, medical history, and lifestyle history. Anthropometric measurements and laboratory tests were also performed.

In this study, we used KNHANES variables related to smoking, namely, the duration of smoking cessation, total smoking period, and average daily smoking amount, to calculate the cumulative amount of smoking. In KNHANES data, ‘smoking’ means cigarette and electronic cigarette smoking. According to Statistics Korea Government Official Work Conference, as of 2018, the smoking prevalence of Koreans, including adolescents, was 22.4% (men, 36.7%; women, 7.5%). [46] A current smoker is defined as a person who has smoked 100 or more cigarettes in their lifetime and continues to smoke [41]. The smoking behavior of participants was divided into never smoker, past smoker, and current smoker. We divided the participants into subgroups on the basis of their previous smoking behavior. In terms of the duration of smoking cessation, the cut-off value of the tertile for former smokers was set so that we could divide them into three groups: <6, 6–17, and ≥17 months. The cumulative smoking amount was derived from calculating the average daily smoking volume (pack) × lifetime smoking period (year) [47]. The subgroups were classified into <5, 5–20, and >20-year-old groups using the cut-off value of the tertile.

In addition, variables such as age, sex, body mass index (BMI), income level, educational level, marital status, alcohol intake, physical activity, obesity, and other variables such as serum total cholesterol, fasting blood glucose, and systolic blood pressure levels through physical examination and laboratory tests were obtained. Alcohol intake was defined as drinking at least one drink per month for the last year, and physical exercise was defined as 150 min of moderate-intensity physical activity, 75 min of high-intensity physical activity, or an equivalent time of mixed moderate- and high-intensity physical activity per week. Obesity was defined as a BMI (calculated by dividing weight (kg) by the square of height (m^2^)) ≥ 25 kg/m^2^. Serum total cholesterol and fasting blood glucose levels were measured using an automatic analyzer (Hitachi 7600, Tokyo, Japan). hs-CRP was measured using immunoturbidimetry (Cobas, Roche, Germany).

The serum hs-CRP level was used as a dependent variable. Values defining elevated serum hs-CRP levels vary slightly among studies. The American Heart Association (AHA), and the Centers for Disease Control and Prevention (CDC) have several findings to determine the concentration of hs-CRP, which represents the risk of developing CVD in the future in healthy adults. They stipulated that the hs-CRP concentration representing the low risk of developing CVD in the future is <1 mg/L, 1–3 mg/L as moderate risk, and >3 mg of hs-CRP as high risk of CVD in the future [8]. On this basis, we defined ≥1 mg/L as elevated serum hs-CRP.

### 2.3. Statistical Analysis

The characteristics of the participants were classified according to elevated serum hs-CRP levels and expressed as means ± standard deviations for continuous variables, and as numbers and percentages for categorical variables. Participant groups were compared using an independent t-test for the continuous variables, and a chi-squared test for the categorical variables.

Multivariate logistic regression analysis was used to analyze the association between each of the duration of smoking cessations and the elevated serum hs-CRP level, and between the cumulative smoking amount during past smoking behaviors and the elevated serum hs-CRP level. Relative risks were estimated in terms of odds ratios (ORs) and 95% confidence intervals (CIs). We adjusted for multiple variables that showed significant associations in the univariate analysis and those with clinical relevance. After calculating the crude ORs (Model 1), Model 2 was adjusted for age, sex, income, education level, marital status, alcohol intake, and physical activity. Model 3 was further adjusted for obesity, systolic blood pressure, fasting blood glucose, and serum total cholesterol levels.

All variables entered into the logistic regression analysis were examined for multicollinearity, and only those with a variance inflation factor <5 were used. All statistical analyses were performed using SPSS version 25 (IBM, Armonk, NY, USA). The level of statistical significance was set at *p* < 0.05, and all *p* values were two-tailed.

## 3. Results

### 3.1. Demographic Characteristics of the Participants

Table 1 shows the demographic characteristics of the participants. The number of participants with elevated serum hs-CRP level was 1124 (20.8% of the total). In terms of sex, the proportion of men in the elevated serum hs-CRP level group was significantly higher (59.7%, *p* < 0.001). 

Compared to the nonelevated serum hs-CRP group, the proportion of the lowest income was higher, and that of the highest income was lower in the elevated serum hs-CRP level group (*p* < 0.01). Serum hs-CRP levels were 0.81 ± 0.61, 0.75 ± 0.56, and 0.68 ± 0.56 mg/L in current, former, and never smokers, respectively (*p* > 0.05, data not shown). The proportion of current and former smokers in the elevated serum hs-CRP level group was 28.5% and 25.9%, respectively. For former smokers, the average duration of smoking cessation was significantly longer in the nonelevated serum hs-CRP level group, with 158.22 ± 4.13 months. In contrast, the average cumulative smoking amount was higher in the elevated serum hs-CRP level group (1.46 ± 0.09 pack-year (PY) (all *p* < 0.05).

The elevated serum hs-CRP level group was associated with a higher proportion of obesity, systolic blood pressure, fasting blood sugar, and serum total cholesterol levels (all *p* < 0.001). 

### 3.2. Association between Duration of Smoking Cessation and Elevated Serum hs-CRP Level

Table 2 shows the ORs and 95% CIs from those with elevated serum hs-CRP levels compared to current smokers after dividing them into three subgroups according to the total former smokers’ duration of smoking cessations. The model was adjusted for age, sex, income, education level, marital status, alcohol intake, physical activity, obesity, systolic blood pressure, fasting blood glucose, and serum total cholesterol level. The adjusted ORs of covariates are also presented (Supplementary Table S1). In the group who had stopped smoking for >17 months, the OR of elevated serum hs-CRP level was 0.73 lower than that of current smokers (95% CI: 0.57–0.92 for Model 3, *p* < 0.01).

### 3.3. Association between Cumulative Smoking Amount and Elevated Serum hs-CRP Level

Table 3 shows the ORs and 95% CIs of those with elevated serum hs-CRP levels compared to current smokers after classifying the cumulative smoking amount of total former smokers into three subgroups. In Model 3, which was adjusted for several factors (age, sex, income, education level, marital status, alcohol intake, physical activity, obesity systolic blood pressure, fasting blood glucose, and serum total cholesterol level), the OR of those with cumulative exposure below 5 PY was 0.71 times lower (95% CI 0.50–0.99, *p* = 0.047), and that of the 5–20PY group was 0.67 times lower (95% CI 0.50–0.92, *p* = 0.012), both compared to current smokers. Those with cumulative exposure >20 PY group showed no substantial reduction.

## 4. Discussion

In this study, we analyzed the association between serum hs-CRP levels of former and current smokers using data from the KNHANES 2016–2018. We confirmed that the odds of elevated serum hs-CRP levels were low in former smokers with a duration of smoking cessation >17 months and a cumulative smoking amount <20 years. Sohn’s cross-sectional study of 1243 former smokers of adult men with the KNHANES 2015–2016 [35] showed a decrease in serum hs-CRP levels in the 10-year and 5–10 quitting groups compared to those who quit smoking <5 years. However, the difference was significant when comparing the <5 years and >10 years groups (OR, 0.64; 95% CI 0.41–0.98, *p* = 0.038). A study by Xiang Qian Lao et al. [48] of Chinese men aged 50–85 years showed that serum hs-CRP concentrations over the period of smoking cessation were elevated in those who quit smoking <5 years compared to current smokers, but began to decline from 5 to 9 years onwards. Concentrations declined to the same extent as that in never smokers in those who quit smoking >20 years. A study by Ohsawa et al. [49], who studied the association between serum hs-CRP and smoking behavior in men aged 40–69 years in Japan also showed lower serum hs-CRP concentrations in the group that quit smoking >5 years than in those who quit smoking <5 years. (0.58 mg/L vs. 0.45 mg/L, *p* < 0.05) Although these studies excluded female smokers, they are in line with the results of this study, which show that longer periods of smoking cessation have a more positive effect on serum hs-CRP level reduction. However, in a longitudinal study by Cecile et al. [50] on changes in inflammatory markers through a 1-year follow-up of smoker and former-smoker groups, quitting smoking did not show a change in serum hs-CRP level. Since there was also a significant decrease in serum hs-CRP level after smoking cessation >17 months in this study, a decrease in serum hs-CRP level may not be observed during a relatively short follow-up. This suggests that at least several months may be required for the inflammatory response from smoking to disappear. 

Moreover, this study also shows no significant odds of elevated hs-CRP levels in former smokers with a cumulative smoking amount >20 pack-years compared with current smokers. This suggests that a spontaneous reduction in serum hs-CRP levels may be difficult to achieve, and elevated serum hs-CRP level is maintained even if heavy smokers quit smoking. A longitudinal study [51] of 975 heavy smokers (>20 pack-years) over the age of 50 who are currently subject to screening for low-dose chest CT found that there was no significant change in serum hs-CRP level in those who quit smoking for more than 1, 3, or 4 years when elevated serum hs-CRP level was defined as 2 mg/L (in order: ORs: 1.21 (95% CI: 0.63–2.33), 1.04 (95% CI: 0.63–1.71), 0.91 (95% CI: 0.45–1.81)). The reason that serum hs-CRP level reduction was not observed in heavy smokers despite long-term follow-up can be assumed, as in the results of our study, as participants were heavy smokers with a high cumulative smoking amount. This hindered reducing serum hs-CRP levels. In a study by Sohn et al. [35] that considered the cumulative smoking amounts of former smokers together, although there was no statistical significance, cumulative smoking amount among former smokers was directly associated with a higher increase in serum hs-CRP levels. In addition, Frohlich et al. [52] reported an association between cumulative smoking amount and serum hs-CRP concentration in smokers who smoked more than 13 cigarettes per day. Therefore, our results, which show a decrease in elevated serum hs-CRP levels when the cumulative amount of smoking was less than 20 pack-years, may explain the dose–response relationship between smoking cessation and CVD and cancer risk.

Smoking is a major risk factor for the inflammation of blood vessel walls, which can be assessed using serum hs-CRP [8]. Smoking induces an inflammatory response in the body through several mechanisms. At the start of smoking, free radicals and harmful substances contained in the smoke stimulate the oral cavity, throat, and tracheal mucosa to directly inflame [23,53]. In the lungs, the largest organ that is mainly exposed to cigarette smoke, cigarette smoke makes lung cells (bronchoalveolar, alveolar epithelial cells, and macrophages of the lung alveoli) release inflammatory cytokines through proinflammatory signaling [54,55,56]. Substances in the smoke lining the walls of peripheral blood vessels can also cause endothelial injury [23]. In addition, the concentration of substances in tobacco increases the number of white blood cells in the peripheral blood [23] and stimulates cell-to-cell adhesion [57]. This facilitates increased numbers of white blood cells to adhere to the vascular endothelium and cause atherosclerotic deterioration [57,58]. These triggered inflammatory responses cause inflammatory cells to secrete proinflammatory cytokines, which attach to the surface of hepatocytes and elevate serum hs-CRP through signaling [30]. Increased serum hs-CRP levels are associated with a series of processes associated with atherosclerosis, future CVD, and cancer; however, when quitting smoking, the influx of these causative agents is cut off, so over time, serum hs-CRP decreases as other inflammatory markers normalize [32].

However, as in this study, if the duration of smoking cessation is short or the cumulative amount of smoking is large, serum hs-CRP levels may not decrease. This may be associated with chronic inflammation and a number of causative diseases [9] linked to rising serum hs-CRP levels and chronic inflammation. Alternatively, as a result of chronic exposure to smoking, the increased hs-CRP level becomes chronic and takes a certain amount of time to normalize despite stopping the exposure to inflammatory agents. In the future, further research is needed to prove causal relationships, and studies are needed on the time and mechanisms for increased serum hs-CRP levels to normalize after quitting smoking. 

This study has some limitations. As this was a cross-sectional study, it was difficult to determine causal relationships. A prospective longitudinal study with a larger sample is required. In addition, because the information was collected with a questionnaire-type response sheet that patients wrote themselves, there may have been errors in the history and disturbances in the accuracy of responses related to smoking behavior. Furthermore, further correction is needed for various factors that elevate serum hs-CRP levels, for example, chronic inflammatory diseases, including autoimmune conditions, inflammatory bowel disease, organ and tissue injury, and medication history [59]. However, KNHANES survey interviews are conducted by well-trained staff to obtain consistent and reliable answers [60], and self-reports are common in population-based studies [61]. Moreover, the reliability of the data and the representation of the population groups were ensured using publicly available data on the country’s largest population. Since it is difficult to generalize the study findings to other countries of regions, further research on subjects of various ethnicities and countries is needed. Furthermore, additional adjustment for various factors that may cause elevation of serum hs-CRP levels is required.

This study is the first to quantitatively investigate the association between serum hs-CRP levels on the basis of the duration of smoking cessation and the cumulative smoking amount of adult former smokers compared to current smokers using nationwide data. This study showed that in former smokers compared to current smokers, there would be a significant decrease in elevated serum hs-CRP levels only 17 months after quitting smoking, and that a decrease in elevated hs-CRP levels would not occur if the cumulative amount of smoking was more than 20 pack-years. 

Our study visualized the benefits of quitting smoking on the basis of evidence as a specific number of smoking periods of more than 17 months and a cumulative smoking amount of less than 20 years, with the hope that it would be able to provide stronger motivation for smokers who are willing to quit, and to guide them in smoking cessation for a long time. In addition, through the results of this study, we can determine the benefits of quitting smoking using patients’ serum hs-CRP levels to show the future risks. Serum hs-CRP is an accessible tool that can be easily examined in hospitalized patients, outpatients, and health checkup centers. A future prospective longitudinal study with a large sample is needed to confirm the benefits of reducing serum hs-CRP level in smoking cessation. As shown in the results of this study, serum hs-CRP can be a useful item for primary care physicians to be more actively involved in motivating patients to quit smoking, to modify their lifestyle, and to care for their chronic disease. 

## 5. Conclusions

In conclusion, a duration of smoking cessation of more than 17 months and a cumulative smoking amount of less than 20 pack years were significantly associated with a decreased risk of elevated serum hs-CRP levels in Korean adults who were former smokers.

## Figures and Tables

**Table 1:** Demographic characteristics according to the elevated serum hs-CRP level * in Korean adults.

Variables	Elevated Serum hs-CRP Level (−) (*n* = 4287)	Elevated Serum hs-CRP Level (+) (*n* = 1124)	*p* Value
Age (years)	50.90 ± 0.14	51.34 ± 0.24	0.087
Male sex (%)	1846 (50.6)	577 (59.7)	<0.001
Income			0.007
Q1 lowest	899 (21.4)	290 (26.1)	
Q2 mid-low	1058 (25.0)	286 (24.9)	
Q3 mid-high	1135 (26.8)	273 (24.9)	
Q4 highest	1189 (26.8)	272 (24.1)	
Education level			0.460
Middle-school graduation or under	879 (18.6)	260 (19.7)	
High-school graduation or higher	3389 (81.4)	863 (80.3)	
Marital status			0.468
Unmarried	195 (5.1)	66 (6.3)	
Married	3711 (86.7)	963 (85.3)	
Divorced or widowed	378 (8.1)	95 (8.3)	
Smoking status			0.003
Current smoker	777 (20.9)	283 (28.5)	
Former smoker	937 (24.8)	258 (25.9)	
Never smoker	2561 (54.3)	582 (45.6)	
Duration of smoking cessation (months for former smokers; values as a categorical variable are presented below)	158.22 ± 4.13	140.45 ± 7.79	0.043
< 6 months	50 (4.9)	15 (6.0)	
6–17 months	51 (5.8)	14 (5.6)	
≥17 months	836 (89.3)	229 (88.4)	
Cumulative smoking amounts (pack-years for former smokers; values as categorical variable are presented below)	12.2 ± 0.05	14.6 ± 0.09	0.024
<5 pack-year	289 (31.3)	73 (26.7)	
5–20 pack-year	399 (43.3)	97 (39.4)	
≥20 pack-year	249 (25.5)	88 (33.8)	
Alcohol intake			0.830
Non drinker	1761 (37.8)	465 (38.2)	
Current drinker	2515 (62.2)	659 (61.8)	
Physical activity ^†^	1913 (45.8)	479 (43.4)	0.215
Obesity ^‡^	1318 (32.2)	568 (50.3)	<0.001
Systolic blood pressure (mmHg)	117.29 ± 0.29	120.59 ± 0.53	<0.001
Fasting blood glucose (mg/dL)	101.08 ± 0.40	108.00 ± 1.04	<0.001
Serum total cholesterol	198.89 ± 0.60	205.85 ± 1.40	<0.001
(mg/dL)			
Serum hs-CRP (mg/L)	0.48 ± 0.04 × 10^−1^	1.65 ± 0.02	<0.001

hs-CRP, high sensitivity C-reactive protein. Data were obtained from the 2016–2018 Korean National Health and Nutrition Examination Survey. *p*-values were calculated using an independent t-test or a chi-squared test. Continuous variables are expressed as means and standard deviations, whereas categorical variables are expressed as numbers and percentages. * Defined as a serum hs-CRP level above 1.0 mg/L. ^†^ Defined as 150 min of moderate-intensity physical activity or 75 min of high-intensity physical activity, or an equivalent time of mixed moderate- and high-intensity physical activity per week. ^‡^ Defined as a body mass index ≥ 25 kg/m^2^.

**Table 2:** Unadjusted and adjusted odds ratios and 95% confidence intervals for the elevated serum hs-CRP level * according to duration of smoking cessation in Korean adults.

Multivariate Model	Model 1 OR (95% CI)	Model 2 OR (95% CI)	Model 3 OR (95% CI)
Current smokers	Reference	Reference	Reference
Former smokers			
Duration of smoking cessation			
<6 months	0.92 (0.47–1.83) *p* = 0.817	0.96 (0.49–1.86) *p* = 0.895	0.90 (0.45–1.79) *p* = 0.758
6–17 months	0.75 (0.39–1.43) *p* = 0.379	0.74 (0.38–1.43)) *p* = 0.370	0.73 (0.38–1.40) *p* = 0.347
≥17 months	0.75 (0.60–0.95) *p* = 0.016	0.75 (0.59–0.95) *p* = 0.018	0.73 (0.57–0.92) *p* = 0.009

hs-CRP, high-sensitivity C-reactive protein; OR, odds ratio; CI, confidence interval. * Defined as a serum hs-CRP level above 1.0 mg/L. Data from the 2016–2018 Korean National Health and Nutrition Examination Survey. Model 1 was crude. Model 2 was adjusted for age, sex, income, educational level, marital status, alcohol intake, and physical activity. Model 3 was adjusted for obesity (defined as a body mass index ≥ 25 kg/m^2^), systolic blood pressure, fasting blood glucose, and total cholesterol, in addition to the variables adjusted in Model 2.

**Table 3:** Unadjusted and adjusted odds ratios and 95% confidence intervals for the elevated serum hs-CRP level * according to cumulative smoking amount in Korean adults.

Multivariate Model	Model 1 OR (95% CI)	Model 2 OR (95% CI)	Model 3 OR (95% CI)
Current smokers	Reference	Reference	Reference
Former smokers			
Cumulative smoking amount			
<5 pack-year	0.65 (0.47–0.91) *p* = 0.013	0.70 (0.50–0.99) *p* = 0.042	0.71 (0.50–0.99) *p* = 0.047
5~20 pack-year	0.69 (0.51–0.94) *p* = 0.020	0.68 (0.50–0.93) *p* = 0.016	0.67 (0.50–0.92) *p* = 0.012
≥20 pack-year	1.01 (0.75–1.37) *p* = 0.936	0.97 (0.71–1.33) *p* = 0.851	0.88 (0.63–1.22) *p* = 0.430

hs-CRP, high-sensitivity C-reactive protein; OR, odds ratio; CI, confidence interval. * Defined as a serum hs-CRP level above 1.0 mg/dL. Data from the 2016–2018 Korean National Health and Nutrition Examination Survey. Model 1 was crude. Model 2 was adjusted for age, sex, income, educational level, marital status, alcohol intake, and physical activity. Model 3 was adjusted for obesity (defined as a body mass index ≥ 25 kg/m^2^), systolic blood pressure, fasting blood glucose, and total cholesterol, in addition to the variables adjusted in Model 2.

## Data Availability

The analyzed datasets during the current study are available in the The KNHANES repository, https://knhanes.kdca.go.kr/knhanes/sub03/sub03_02_05.do (accessed on 2 February 2022). KNHANES is a nationwide population-based survey conducted by the Korean Ministry of Health and Welfare, and the Division of Chronic Disease Surveillance of the Korean Centers for Disease Control and Prevention. All data are fully available without restriction. All data files are available from the KNHANES database.

## Supplementary Materials

The following supporting information can be downloaded at: https://www.mdpi.com/article/10.3390/toxics10090533/s1. Table S1: unadjusted and adjusted odds ratios and 95% confidence intervals of covariates for the elevated serum hs-CRP level * in Korean adults from KNHANES 2016 to 2018.
