# Supplementary material for: Association of Duration of Smoking Cessation or Cumulative Smoking Amount with Serum hs-CRP Level in Korean Adults: A Nationwide Population-Based Cross-Sectional Study
Source: Toxics. 2022 Sep 10;10(9):533. doi: 10.3390/toxics10090533 (PMC9501822; doi:10.3390/toxics10090533)
Supplement: Supplementary file 1 [file toxics-10-00533-s001.zip › toxics-1832540-supplementary.pdf]

Supplementary table S1. Unadjusted and adjusted odds ratios and 95% confidence intervals of covariates for the elevated serum hs-CRP level* in Korean adults from KNHANES 2016 to 2018.

Variables	Adjusted OR (95% CI)	P value
Age (year)	1.01 (1.00 – 1.02)	0.231
Sex		0.009
Male	1.27 (1.06 – 1.51)	
Female (reference)	1	
Income		0.155
Q1 lowest	1.29 (1.02 – 1.64)	
Q2 mid-low	1.09 (0.87 – 1.36)	
Q3 mid-high	1.02 (0.81 –1.28)	
Q4 highest (reference)	1	
Education		0.452
Middle school graduation or under	0.92 (0.73 – 1.15)	
High school graduation or higher (reference)	1	
Marital status		0.616
Unmarried (reference)	1	
Married	0.84 (0.60 – 1.19)	
Divorced or widowed	0.84 (0.55 – 1.30)	
Alcohol intake		0.071
Non drinker (reference)	0.86 (0.73 – 1.01)	
Current drinker	1	
Physical activity		0.203
No	1.11 (0.95 – 1.30)	
Yes (reference)	1	
Obesity†		<0.001
Yes	1.90 (1.61 – 2.23)	
No (reference)	1	
Systolic blood pressure (mmHg)	1.006 (1.001 – 1.011)	0.031
Fasting blood glucose (mg/dL)	1.007 (1.004 – 1.010)	<0.001
Serum total cholesterol (mg/dL)	1.005 (1.003 – 1.007)	<0.001

hs-CRP, high-sensitivity C-reactive protein; KNHANES, Korea National Health and Nutrition Examination Survey; OR, odds ratio; CI, confidence interval.

*Defined as a serum hs-CRP level above 1.0 mg/L.

†Defined as body mass index ≥25 kg/m2

Adjusted for age (continuous variable), sex, income, educational level, marital status, alcohol intake, physical activity, obesity, systolic blood pressure (continuous variable), fasting blood glucose (continuous variable), and serum total cholesterol (continuous variable).
